# Characterisation of mobile genetic elements in *Mycoplasma hominis* with the description of ICEHo-II, a variant mycoplasma integrative and conjugative element

**DOI:** 10.1186/s13100-020-00225-9

**Published:** 2020-11-07

**Authors:** Birgit Henrich, Stephanie Hammerlage, Sebastian Scharf, Diana Haberhausen, Ursula Fürnkranz, Karl Köhrer, Lena Peitzmann, Pier Luigi Fiori, Joachim Spergser, Klaus Pfeffer, Alexander T. Dilthey

**Affiliations:** 1 Institute of Med. Microbiology and Hospital Hygiene of the Heinrich-Heine-University Duesseldorf, Duesseldorf, Germany; 2 Department of Haematology, Oncology and Clinical Immunology, Medical Faculty, University of Duesseldorf, Duesseldorf, Germany; 3 Institute for Specific Prophylaxis and Tropical Medicine, Centre for Pathophysiology, Immunology and Infectiology, Medical University of Vienna, Vienna, Austria; 4 Biological and Medical Research Centre (BMFZ) of the Heinrich-Heine-University Duesseldorf, Duesseldorf, Germany; 5 Department of Biomedical Sciences, University of Sassari, Sassari, Italy; 6 Institute of Microbiology, University of Veterinary Medicine Vienna, Vienna, Austria; 7 Institute of Medical Statistics and Computational Biology, University of Cologne, Cologne, Germany; 8 Cologne Excellence Cluster on Cellular Stress Responses in Aging-Associated Diseases (CECAD), University of Cologne, Cologne, Germany

**Keywords:** Mobile genetic element, Mycoplasma, *M. hominis*, Nanopore sequencing

## Abstract

**Background:**

Mobile genetic elements are found in genomes throughout the microbial world, mediating genome plasticity and important prokaryotic phenotypes. Even the cell wall-less mycoplasmas, which are known to harbour a minimal set of genes, seem to accumulate mobile genetic elements. In *Mycoplasma hominis*, a facultative pathogen of the human urogenital tract and an inherently very heterogeneous species, four different MGE-classes had been detected until now: insertion sequence ISMhom-1, prophage MHoV-1, a tetracycline resistance mediating transposon, and ICEHo, a species-specific variant of a mycoplasma integrative and conjugative element encoding a T4SS secretion system (termed MICE).

**Results:**

To characterize the prevalence of these MGEs, genomes of 23 *M. hominis* isolates were assembled using whole genome sequencing and bioinformatically analysed for the presence of mobile genetic elements. In addition to the previously described MGEs, a new ICEHo variant was found, which we designate ICEHo-II. Of 15 ICEHo-II genes, five are common MICE genes; eight are unique to ICEHo-II; and two represent a duplication of a gene also present in ICEHo-I. In 150 *M. hominis* isolates and based on a screening PCR, prevalence of ICEHo-I was 40.7%; of ICEHo-II, 28.7%; and of both elements, 15.3%. Activity of ICEHo-I and -II was demonstrated by detection of circularized extrachromosomal forms of the elements through PCR and subsequent Sanger sequencing.

**Conclusions:**

Nanopore sequencing enabled the identification of mobile genetic elements and of ICEHo-II, a novel MICE element of *M. hominis*, whose phenotypic impact and potential impact on pathogenicity can now be elucidated.

**Supplementary Information:**

The online version contains supplementary material available at 10.1186/s13100-020-00225-9.

## Background

*Mycoplasma hominis* is a facultative pathogen of the human urogenital tract and associated with bacterial vaginosis, pelvic inflammatory disease, septic arthritis, preterm birth or even neonatal meningitis [1–3]. The factors accounting for the pathogenic potential of this heterogeneous species with the second smallest genome described so far are not fully understood. Several studies were conducted to characterize host-pathogen interactions in vitro [4–6] and in vivo [7–9], including microarray-based characterization of host [10] and pathogen [11] transcriptome changes in *M. hominis* infection. With increasing numbers of completely resolved *M. hominis* genomes (20 at the time of writing; https://www.ncbi.nlm.nih.gov/genome/), however, it became increasingly clear that mobile genetic elements, such as of MhoV-1 [12], ISMhom-1 [13], the *tet(M)*-carrying transposon [14], and the recently detected ICEHo element [15], significantly contribute to genomic plasticity of *M. hominis* [16].

The present study was conducted to elucidate the presence and prevalence of mobile genetic elements in selected clinical strains of *M. hominis.* To ensure the correct resolution and localization of MGE-associated genomic repeats, a Nanopore-based long-read sequencing approach was combined with an Illumina-based assembly polishing strategy.

## Results

### Generation of high-quality assemblies of 11 *M. hominis* strains

A hybrid approach combining short- and long-read sequencing data (Table 1) was used to generate high-quality assemblies of 11 isolates of *M. hominis*. Briefly, the Oxford Nanopore and Pacific Biosciences technologies were used to generate ≥500X of long-read sequencing data for each isolate genome; these data were assembled using Canu [17] or HGAP [18] and polished using ≥100X of short-read Illumina sequencing data for each sample. All assemblies were manually inspected for quality. A full description of the sequencing and assembly process is given in the Methods section. Genome lengths of all 11 isolates were larger than that of type strain PG21 (665 kbp [19]), ranging from 673 kbp (SS25) to 780 kbp (FBG); the number of annotated genes, predicted by Prokka [20], ranged from 580 (SS25) to 680 genes (FBG). Two additional publicly available genome sequences were also incorporated into the analysis (TO0613 and PL5).

**Table 1:** Whole-genome sequencing of 23 *M. hominis* strains

Strain	Short-read sequencing			Long-read sequencing				
	Protocol	Generated data (Mb)	Est. coverage (X)	Technology	Kit	Generated data (Mb)	Est. coverage (X)	Median read length/kb
FBG	2 × 300	1612	2067	Nanopore	SQK-RAD003	3542	4541	2.5
8958	2 × 300	157	231	Nanopore	SQK-RAD003	657	966	4.5
2539	2 × 300	2344	3119	Nanopore	SQK-LSK108	384	511	12.6
A136	2 × 300	240	344	Nanopore	EXP-NBD103 + SQK-LSK108	707	1016	13.6
SP2565	2 × 300	205	287	Nanopore		1092	1533	10.5
475	2 × 300	184	257	Nanopore		638	889	5.3
SS10	2 × 300	1648	2353	Nanopore		756	1080	5.7
SS25	2 × 300	1865	2772	Nanopore		732	1088	8.3
VO31120	2 × 250	89	130	Nanopore		728	1069	6.3
SP10291	2 × 250	80	106	Nanopore		562	749	4.4
SP3615	2 × 250	99	138	PacBio	SMRTbell Template Prep Kit 1.0 + Sequel Binding and Internal Control Kit 2.1	7447	10,401	2.9
727 J				Nanopore	EXP-NBD103 + SQK-LSK108	17	21	5.2
942 J				Nanopore		38	47	44.1
2740				Nanopore		15	16	4.1
7388VA				Nanopore		39	47	41.2
7447VA				Nanopore		59	66	1.7
10936VA				Nanopore		53	66	3.0
12256 U				Nanopore		35	42	14.5
14352VA				Nanopore		37	47	32.3
16753				Nanopore		7	9	4.4
18847				Nanopore		38	49	45.7
19791				Nanopore		44	52	4.6
21127VA				Nanopore		38	44	42.4

### Detection of mobile genetic elements (MGE) in selected *M. hominis* strains

**Fig. 1:**
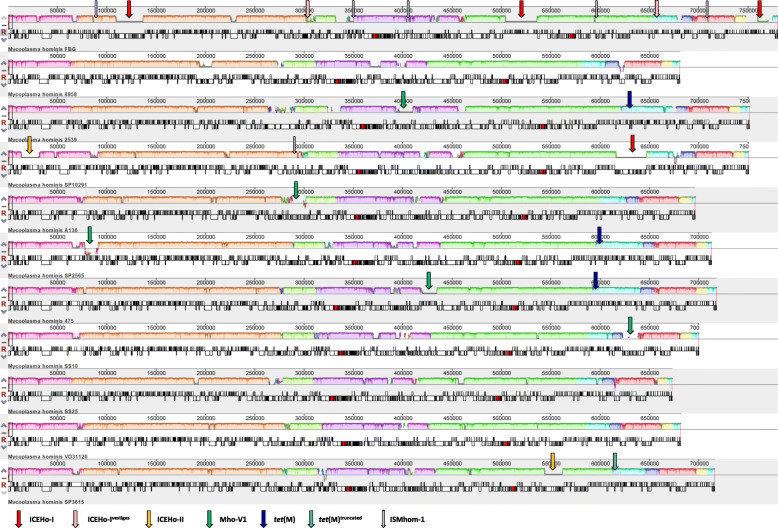
Mauve alignment of *M. hominis* genomes. In Mauve progressive alignment of genomes of *M. hominis* strains FBG, 8958, 2539, SP10291, A136, SP2565, 475, SS10, SS25, VO31120 and SP3615 the FBG genome served as a reference. Regions with the same colour represent locally collinear blocks without rearrangement of the homologous backbone sequences. Open reading frames of both strands are depicted below with rRNA genes in red. Local positions of MGE are marked above genomes by vertical arrows in specific colouring: ICEHo-I in red, ICEHo-I vestiges in pink, ICEHo-II in yellow, MHoV-1 in green, *tet*(M) in dark blue, truncated *tet*(M) in light blue and ISMhom-1 in light purple

The online software tool Mauve [21] was used for genome alignments (Fig. 1) illustrating homologous regions by colour. Thus, larger isolate-specific regions of gene gain were evident by blocks of zero similarity (e.g. uncoloured sections) and classified as putative mobile genetic elements (MGE).

Four different classes of MGE were characterized in the *M. hominis* genomes: i) insertion sequence ISMhom-1, first described in 2008 [13], ii) prophage MHoV-1 [12], iii) a tetracycline resistance mediating transposon [14], and iv) ICEHo-I and -II, two *M. hominis*-specific variants of MICE, a mycoplasma integrative and conjugative element [22], of which ICEHo-I corresponds to ICEHo recently published [15]. All MGE insertion sites are shown in Table 2 and visualized in Fig. 1. We detected between 0 (isolates 8958, SS25, and VO31120) and 8 MGEs (isolate FBG) per genome; of note, the three isolates in which no MGEs were detected had the smallest genome sizes.

**Table 2:** Presence and genomic position of mobile genetic elements

Strain	Genome / bp	ISMhom-1^a^ / bp x – bp y	MHoV-1^b^ /bp x – bp y	*tet*(M)^c^ / bp x – bp y	ICEHo-I ^d^ / bp x – bp y	ICEHo-II ^e^ / bp x – bp y
**FBG**	780,024	85,753–87,012 349,147–350,406 404,580–403,321 593,967–595,226 708,740–709,999	–	–	136,576–109,789 508,629–535,415 747,727–774,523 307,652 – 309385^f^ (675938–675,828) ^g^	–
**8958**	680,851	–	–	–	–	–
**2539**	751,326	–	407,989–392,742	619,357–644,618	–	–
**SP10291**	750,518	293,110–294,369	–	–	646,629–616,362	13,153–31,485
**A136**	696,338	–	301,276–286,023	–	–	–
**SP2565**	712,781	–	91,014–75,747	593,982–619,248	–	–
**475**	717,789	–	419,145–434,419	590,550–615,528	–	–
**SS10**	700,146		637,906–622,632	–	–	–
**SS25**	672,843	–	–	–	–	–
**VO31120**	681,374	–	–	–	–	–
**SP3615**	715,990	–	–	613,432–620,439 ^h^	–	543,628–561,978
TO0613^i^	766,228	–	694,774–710,019	–	49,522–18,905 310,644–280,027	–
PL5	767,767 (JRXA01) ^**j**^	–	–	74,216–99,476(_000009.1) ^**j**^	[89,704–90,264] – [1950–3140] (_000010.1) ^**j**^ (_000001.1) [1814–1029] – [18,377–17,187] (_000005.1) (_000004.1)	–

MGE elements used in BLAST analysis: ^**a**^ ISMhom-1 (acc.-no. dq973625); ^b^ MHoV-1 prophage region from *rep*B to *exi*S (acc.- no. CP009652; bp 596,991-bp 581,744); ^c^ *tet*(M) of SPROTT (acc.- no. CP011538; bp 573,817–599,077); ^d^ ICEHo-I region of FBG (CDS1 to CDS22, bp 508,629 – bp 535,415); ^e^ ICEHo-II region of SP3615 (CDS1 to CDS22, bp 543,628 – bp 561,978); ^f^ICEHo-I vestige (corresponding FBG ICEHo-I-2; bp 508,629 – bp 510,361); ^g^ ICEHo-I vestige (corresponding to FBG ICEHo-I-2 untranslated region 535,715–535,827); ^h^ truncated *tet*(M) transposon corresponding to bp 577,017 - bp 584,024 of SPROTT; accession numbers of ^i^ TO0613 genome (acc.-no CP033021.1) and ^j^ Pl5 contigs (JRXA01_000001.1 to JRXA01_000010.1)

### ISMhom-1

ISMhom-1 (1.26 kb) was found in two isolates; isolate SP10291 contained one copy, and isolate FBG carried five copies. ISMhom-1 was highly conserved in sequence, carrying an open reading frame similar to transposase gene *tnp*A of the IS30 family [13], which was flanked by a nontranslated region (108 bp on the 5′ end and 140 bp 3′) with terminal inverted repeats of 27 bp. Generation of inverted repeats by IS elements was first described for an IS30-type insertion element of *M. fermentans* [23]. ISMhom-1 insertion positions included un-translated regions (FBG ISMhom-1_1, ISMhom-1_2, ISMhom-1_3, and SP10291) and the 3′ ends of the annotated genes BHBFJMJE_00532 and BHBFJMJE_00625 (FBG ISMhom-1_4 and ISMhom-1_5, respectively). The concomitant generation of insertion site-specific inverted repeats resulted in integrity of both ORFs (Table 3).

**Table 3:** Integration sites of mobile genetic elements (MGE)

MGE	Gene / *contig*^**a**^	PG21-homologue^**b**^	Gene product	Gene length (nt)	Insertion site in gene (nt)	DR / *IR* ^c^
**FBG_ISMhom-1_1**	dBHBFJMJE_00070^rc d^	IGR ^e^ (dMHO_0640)	–	–	–	*AAAATA*
**FBG_ISMhom-1_2**	dBHBFJMJE_00328 ^d^	IGR ^e^ (dMHO_2560)	–	–	–	*ATATTT*
**FBG_ISMhom-1_3**	dBHBFJMJE_00386 ^d^	IGR ^e^ (dMHO_3070)	–	–	–	*TAAATTATTTTGGGT*
**FBG_ISMhom-1_4**	BHBFJMJE_00532^rc^	MHO_4140^rc^	put. HAD hydrolase	807	807	***AAATAA***^*f*^
**FBG_ISMhom-1_5**	BHBFJMJE_00625^rc^	MHO_5020^rc^	put. Sporulation transcription regulator WhiA	828	828	***AAATAG****GC*^*f*^
**SP10291_ISMhom-1**	dHPAMDCMO_00271 ^d^	IGR ^e^ (dMHO_2210)	–	–	–	*TGTGCTTTTT*
**2539_MHoV1**	KLHMLDFE_00358	MHO_3090	hypothetical protein	1134	808	ATTTTTAT / ATTTTTTT
**A136_MHoV1**	dMNIFKBBE_00264 ^d^	IGR ^e^ (dMHO_2260^rc^)	–	–	–	TTTTTTTT / CTTTTTTT
**SP2565_MHoV1**	dHHAOGLDO_00056 ^d^	IGR ^e^ (dMHO_0530)	–	–	–	ATTTTTATA / ATTTTTCTA
**475_MHoV1**	dMOHCKDGE_00391 ^d^	IGR ^e^ (dMHO_3470)	–	–	–	TTTTTT / CTTTTT
**SS10_MHoV1**	MFOAEKDO_00548	MHO_4930	hypothetical protein	903	867	CTTTTT (866–867) ^g^
**TO0613_MHoV1**	dKN71_002970 ^d^	IGR ^e^ (dMHO_4930)	–	–	–	ATTTTTA
**FBG_ICEHo-I-1**^rc^	BHBFJMJE_00092	MHO_0820	hypothetical protein	651	394	AAATAATA (387–394) ^g^
**FBG_ICEHo-I-2**	BHBFJMJE_00459	MHO_3720	P75 precursor	1950	1950 + 1^h^	CAAATAAA/AATCTTT ^i^ (1323–1950) ^g^
**FBG_ICEHo-I-3**	BHBFJMJE_00651	MHO_5300	conserved hypothetical protein	432	432 + 2^h^	**AAATAA**AA^*f*^ (427–432) ^g^
**FBG_ICEHo-I-4 vestige**	BHBFJMJE_00287	MHO_2250	hypothetical protein	663	44	
**FBG_ICEHo-I-5 vestige**^rc^	dBHBFJMJE_00595 ^d^	IGR ^e^ (dMHO_4730)	*–*	–	–	
**SP10291_ICEHo-I**	dHDENHCDK_00935 ^d^	IGR ^e^ (dMHO_4550)	–	–	–	AAATTTTT
**TO0613_ICEHo-I-1**^**rc**^	dKN71_000220 ^d^	IGR ^e^ (dMHO_0170^rc^)	–	–	–	TTTAAAAT
**TO0613_ICEHo-I-2**^**rc**^	KN71_001280	MHO_2080	conserved hypothetical lipoprotein	1734	1407	**TGGAAAT** (1401–1407) ^g^
**PL5_ICEHo-I-1**	*JRXA01000010.1* *JRX01A000001.1*	MHO_0120	type-III restriction enzyme	2453	853	**TTTTTAAA** (846–853) ^g^
**PL5_ICEHo-I-2**	*JRXA01000004.1* *JRXA01000005.1*	MHO_1960	hypothetical protein	369	4	AAATAAAG (4–12) ^g^
**SP3615_ICEHo-II**	dKGPEAEHF_00485^rc d^	IGR ^e^ (d*dpn*A)	methylase DpnIIB	–	–	TAATATTA
**SP10291_ICEHo-II**	HPAMDCMO_00015	MHO_0130	site-specific DNA methyl-transferase (DEAD/DEAH box helicase family protein)	1200	1152	**AAATCTTT** (1145–1152) ^g^

^a^Contigs only shown for strain PL5, due to assembly fragmentation; printed in italics; ^b^ MHO genes of PG21 according to acc.-no: FP236530.1; ^c^ DR = direct repeat; *IR = inverted repeat*; ^d^ dgene_X = insertion site downstream of gene X; ^rc^ = gene on reverse complementary strand; ^e^ IGR (= intergenic region) downstream of MHO-homologous gene X (dMHO_X); ^*f*^ downstream DR identical to 3′ end of the gene, protein encoding regions in bold; ^*g*^ DR region in the affected gene (nt x to nt y); ^h^ + 1 /+ 2 = direct repeat terminates 1 nt / 2 nt downstream of gene x resulting in downstream DR containing 3′ end of the gene; ^i^ no DR, but duplicated region of p75 (nt 1323–1950)

### Prophage MHoV-1

Prophage MHoV-1 was detected in six isolates (five de novo assembled genomes and one publicly available genome, TO0613). Presence and sequence of genes (from *rep*B to *exi*S, i.e. spanning the complete MHoV-1 element as defined by [12], and terminated by indirect (IR; AAAGTCCC) repeats of the phage) were highly conserved across the de novo assembled genomes (Table 3). The respective prophage region in TO0613 was structurally consistent with the de novo assembled sequences; several annotated TO0613 genes, however, were disrupted, suggesting a potential assembly problem in the published MiSeq-based assembly of TO0613. No systematic patterns of MHoV-1 integration positions were observed (Table 3). In four cases (strains A136, SP2565, 475 and TO0613), MHoV-1 integrated into intergenic regions; in two cases (strains 2539 and SS10), into open reading frames encoding hypothetical genes of unknown function, leading to premature disruption of the predicted hypothetical genes.

### *tet*(M)-harbouring transposon

**Fig. 2:**
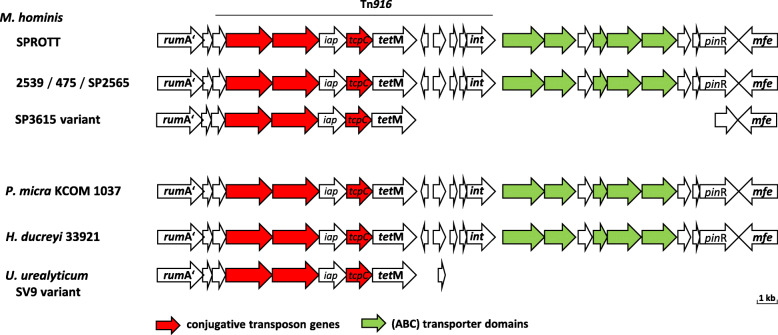
Organisation of *tet(M)* transposons. An identical organisation of genes was found in *tet(M)* transposons of *M. hominis* strains SPROTT (acc.- no. CP011538.1; nt 573,817-599,077), 2539, 475, SP2565, *Parvimonas micra* strain KCOM 1037 (CP031971.1; nt 249,283-277,971)) and *H. ducreyi* strain 33,921 (CP011228.1; nt 678,636–662,601 and nt 1,365,672-1,378,984). The region comprising the truncated Tn916 unit (13.3 kb) is marked by a solid line. Truncated variants with loss of the *int* gene were found in *M. hominis* strain SP3615 (nt 609,362-623,230) and *U. urealyticum* serovar 9 str. ATCC 33175 (AAYQ02000002.1; nt 59,407-45,316))

A *tet*(M)-harbouring transposon of 25 kb length, mediating tetracycline resistance, was detected in four *M. hominis* strains (2539, 475, SP2565, and PL5). The transposon was highly conserved in gene organisation (see Fig. 2) and sequence (> 94% nucleotide identity), and comprised a 13.3 kb region homologous to transposon Tn916 [14]. Insertion sites of the *tet*(M)-harbouring transposon were highly conserved, targeting the 3′ end of the *rumA* gene and leading to RumA C-terminal extension, consistent with findings in strain SPROTT [14], in which a homologous full-length transposon is also present (Fig. 2). Truncated versions of the element were found in strain SP3615 (encompassing conjugative transposon genes but missing integrase gene *int*), as well as in *Ureaplasma urealyticum*, serovar 9 (Fig. 2). The functional relevance of these truncations remains unclear. Further BLAST analyses identified a homologous transposon in *Parvimonas micra* (> 87% nucleotide identity), a pathogen which is commonly found in the oral cavity or gastrointestinal tract [24], and two homologous regions in *Haemophilus ducreyi* strain 33,921 (acc.-no. CP011228.1), covering the entire transposon with > 87% nucleotide identity.

### Mycoplasma integrative and conjugative elements

Two different MICE variants, ICEHo-I and ICEHo-II, were detected in the *M. hominis* genomes sequenced in this study. The first of these, ICEHo-I, was previously characterized by Meygret el al [15]. and named ICEHo.

### Integrative and conjugative element ICEHo-I

ICEHo-I was detected in four isolate genomes, with copy numbers varying between 1 (SP10291), 2 (strains TO0613 and PL5), and 3 (strain FBG); the genomic locations and features of the integration sites of ICEHo-I are summarized in Tables 2 and 3.

**Fig. 3:**
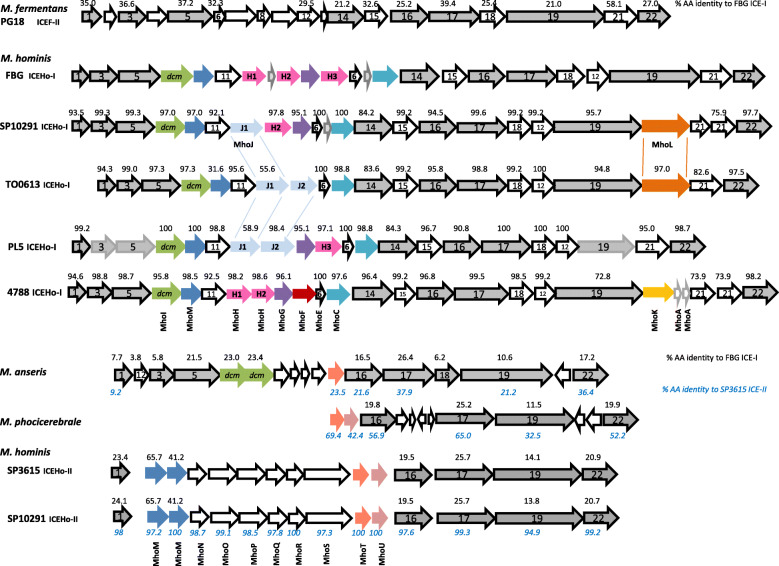
Structural organisation of ICEHo elements. Protein sequences of annotated MICE genes were obtained from NCBI for *M. fermentans* PG18 ICEF-II (MBIO_0551–0567 and MBIO_0555–5 (inserted by hand; nt 616,241 to 616,429 of AP009608.1)), *M. hominis* strains FBG ICEHo-I (BHBFJME_00460–00482), SP10291 ICEHo-I (HPAMDCMO_00562–00540) and ICEHo-II (HPAMDCMO_00016–00030), SP3615 ICEHo-II (KGPEAHF_00500–00483), TO0613 ICEHo-I (KN71_RS02000–01900), PL5 ICEHo-I (V136_RS01075/_03225–03305/VE10_RS00015), 4788_ICEHo-I (EVJ69_RS02290–02175), *M. anseris* ICEHo-I/II (DP065_01405–01485) and *M. phocicerebrale* ICEHo-II (DMC14_02545–02600) and analysed by multiple protein sequence alignments. Sequence identities of homologous proteins, estimated by ClustalW, are shown above the encoding ORFs in relation to FBG ICEHo-I, and below in relation to SP3615 ICEHo-II. Percent amino acid identities of MhoJ1 and MhoJ2 of ICEHo-I were calculated with respect to MhoJ1 of SP10291 ICEHo-I and MhoJ2 of TO0613 and respectively marked by lines. Incomplete recovery of CDS3, CDS5 and CDS19 of strain PL5, which did not enable calculation of homologies, is indicated by transparent framing

ICEHo-I carried a set of 13 MICE core genes as defined in an analysis of MICE of *M. fermentans* M64 and *M. agalactiae* 5632 [22]. MICE core genes exhibited a high degree of conservation across the assembled *M. hominis* genomes; inter-strain homologies of the MICE core proteins ranged from 76 to 100% with respect to strain FBG (Fig. 3). Inter-species homologies, by contrast, were lower; for example, protein homologies with respect to MICEF-II of *M. fermentans* [22] ranged from 21% (CDS19) to 58% (CDS21). Of note, the set of MICE core genes present in ICEHo-I included CDS6. In the original description of ICEHo-I [15], a highly homologous gene (EVJ69_RS02240 in strain 4788; 100% amino acid identity) had been classified as a non-core MICE gene [15]; identification with CDS6, however, was justified by 32.3% amino acid identity and 53.2% amino acid similarity to ICEF-ORF6 of *M. fermentans* (Additional file 1).

An analysis of MICE non-core (i.e. cargo) genes in ICEHo-I showed that the genes *dcm*, MhoM, MhoE, and MhoC were always present at a single copy, and their relative position was conserved (Fig. 3). MhoH, MhoG, MhoF, and MhoJ were consistently located between CDS11 and MhoE, and their copy number was variable (ranging from 0 to 1 for MhoG and MhoF; from 0 to 2, for MhoJ; and from 0 and 3, for MhoH). MhoA, MhoK, and MhoL were located between CDS19 and CDS22 and varied in copy number between 0 and 1 (MhoL and MhoK) and 0 and 2 (MhoA). In a phylogenetic analysis of MhoH, MhoJ, and MhoF, MhoF of strain 4788 clustered with MhoH (Additional file 2), demonstrating that MhoH and MhoF are closely related.

ICEHo-I untranslated regions (210 bp upstream of CDS1 and 413 bp downstream of CDS22) were highly conserved and terminated by an inverted repeat. ICEHo-I integration into host genomes resulted in the generation of direct repeats (Table 3). In two instances, integration was associated with a premature stop of translation, affecting a hypothetical protein (strain FBG; at nucleotide 394/651 of the MHO-0820-homologous BHBFJMJE_00092) and a lipoprotein (strain TO0613; at nucleotide 1407/1734 of the MHO-2080 homologue). In strain PL5, analysis of insertion sites was limited by incomplete genome resolution, but BLAST analysis suggested an insertion into the MHO-0120- and MHO-1960-homologous genes, putatively encoding a type III restriction enzyme and a hypothetical protein, respectively. In strain FBG, integration of ICEHo-I-2 was associated with a large duplication within the P75 precursor gene resulting in an upstream intact P75-precursor gene (nucleotide 1–1950) and a downstream remnant (nucleotide 1323–1950).

The three complete copies of ICEHo-I in strain FBG exhibited a high degree of conservation and differed by only four nucleotides, associated with a single amino acid exchange in CDS14 of ICEHo-I_3 (^Asn^485^Ile^). In addition to three complete copies of ICEHo-I, strain FBG also harboured two ICEHo-I vestiges (FBG ICEHo-I-4 and -5; see Fig. 1 and Table 3).

### Integrative and conjugative element ICEHo-II

The detection of two additional regions of zero similarity with respect to the other *M. hominis* genomes in strains SP3615 and SP10291 (highlighted in Fig. 1) led to the discovery of another mycoplasma integrative and conjugative element, referred to as ICEHo-II. ICEHo-II was conserved in length (~18kbp) and sequence of 15 open reading frames (Fig. 3; 94.9–100% AA identity).

Protein homology analyses classified five of the open reading frames as MICE-core genes CDS-1, − 16, − 17, − 19, and − 22, with homologies of the encoded proteins to the respective ICEHo-I proteins of FBG-ICEHo-I ranging from 14.1% (CDS19) to 25.7% (CDS17). Of the ICEHo-II cargo gene encoded proteins (MhoM to MhoU), only protein MhoM, duplicated in ICEHo-II, was also found in ICEHo-I with 30–50% AA identity. A phylogenetic analysis showed that MhoM generally clustered distinctly from CDS11 into ICEHo-I- or –II-specific branches; except for ICEHo-I MhoM protein of TO0613, which was phylogenetically positioned between ICEHo-II MhoM and CDS11 (Additional file 3).

ICEHo-II untranslated regions (207 bp upstream of CDS1 and 210 bp downstream of CDS 22) were terminated by inverted repeats (IRL: GGCCGTGTAAAAAATATAAGGAAT and IRR: ATTCCTTTAATAATAAACACGACC). In strain SP10291, ICEHo-II insertion led to a premature stop in the MHO-0130-homologous gene, putatively encoding a site-specific DNA methyltransferase belonging to the DEAD/DEAH box helicase family (see Table 3). In strain SP3615, ICEHo-II was reversely inserted between the MHO-4180- and MHO-4190-homologous genes, and three additional genes (KGPEAEHF_0485 to KGPEAEHF_0483) were detected between MHO_4180 and ICEHo-II. A Phyre^2^ analysis of these genes showed homologies to two methyltransferases (KGPEAEHF_0485 and KGPEAEHF_0486) and a *S. pneumoniae* endonuclease encoded in the DpnII gene cassette (KGPEAEHF_0483).

A BLAST analysis identified ICEHo-II-homologous regions in other mycoplasma species, *M. phocicerebrale* [25] and *M. anseris* [26] (Fig. 3). In the seal pathogen *M. phocicerebrale* a truncated ICEHo-II region was detected, extending from gene MhoT to CDS22. In the duck and goose pathogen *M. anseris* a hybrid ICEHo element was found, carrying the ICEHo-I- homologous MICE genes CDS3, − 5, − 12, − 18, and *dcm,* and the ICEHo-II homologous MICE genes CDS1, − 16, − 17, − 19, − 22, and MhoT, suggesting a common ancestor or a product of recombination of both ICEHo elements.

### Prevalence of ICEHo-I and ICEHo-II elements

Using a Real time PCR (qPCR) screening approach targeting ICEHo-I and -II-specific small gene fragments, 150 isolates from the *M. hominis* strain collection of our institute were tested for the presence of ICEHo elements (see Methods). For ICEHo-I, 57.3% of the *M. hominis* strains (86/150) were rated as unambiguously ICEHo-I-negative, and 28% (42/150) were classified as unambiguously ICEHo-I-positive. Of the remaining 22 isolates with ambiguous ICEHo-I-specific probe detection results, 19 isolates were rated as ICEHo-I positive, yielding an overall ICEHo-I detection rate of 40.7% (61/150). For ICEHo-II, 28.7% of the *M. hominis* strains (43/150) were tested ICEHo-II-positive; including 15.3% strains (23/150) also positive for ICEHo-I.

To verify the accuracy of the qPCR screen, additional Nanopore long-read sequencing data were generated on 12 isolates (Table 1), draft de novo assembly was carried out, and ICEHo-I and -II positions in the de novo assemblies were determined. ICEHo detection results and genomic locations in the draft de novo assembled genomes are summarized in Additional file 4. The determined ICEHo-I and -II copy number counts agreed with the screening-based results for each evaluated isolate, confirming the accuracy of the qPCR screen. Variability in ICEHo-I structure, as already observed in the set of genomes assembled to high quality, was also found in the newly sequenced isolates; by contrast, the structure of ICEHo-II was found to be highly conserved (Additional file 4).

### MGE co-occurrence analysis

MGE copy numbers were tabulated across the assembled genomes (Table 4) and statistical tests were carried out to assess the evidence for non-random co-occurrence of different MGEs, using the Chi-Square test to detect associations at the level of presence and absence and Spearman’s rank correlation test to detect associations at the level of MGE multiplicity. No statistically significant association at *p* = 0.05 was found between the presence or multiplicity of ICEHo-II in a given strain and presence of any other MGE; the lowest *p*-values were achieved for MhoV-1 being present more often in the absence of ICEHo-I (*p* = 0.066) and ISMhom-1 only occurring when ICEHo-I was present (*p* = 0.096).

**Table 4:** MGE copy number in assembled *M. hominis* genomes

Isolate	ISMhom1^a^	MhoV1^b^	*tet*(M) transposon^c^	ICEHo-I^d^	ICEHo-II^e^
FBG	5	0	0	3	0
8958	0	0	0	0	0
2539	0	1	1	0	0
SP10291	1	0	0	1	1
A136	0	1	0	0	0
SP2565	0	1	1	0	0
475	0	1	1	0	0
SS10	0	1	0	0	0
SS25	0	0	0	0	0
VO31120	0	0	0	0	0
SP3615	0	0	1	0	1
TO0613	0	1	0	2	0
PL5	0	0	1	2	0
18847	0	0	0	1	0
21127	0	0	1	3	0
7388	0	1	1	0	0
727 J	0	0	0	0	1
7447VA	0	1	0	0	1
2740	0	1	0	0	2
16753	0	1	1	0	2
942 J	0	1	0	1	1
10936	0	0	1	1	1
12256	0	0	0	1	2
14352	0	0	0	1	1
19791	0	1	1	1	2

MGEs were detected using pairwise sequence alignments between the assembly and MGE query sequences ^a^ ISMhom-1, query sequence acc.-no. dq973625, detection threshold: > 80% identity; ^b^ MHoV-1 prophage, query sequences CP009652:581744–584,733 (covering *rep*B) and CP009652:596991–598,991 (covering *exi*S), detection threshold: > 80% identity for both query sequences; ^c^ *tet*(M), query sequence CP011538: 573817–599,077 (truncated *tet*(M) transposon, query sequence CP011538:577017–584,024) (derived from SPROTT), detection threshold: > 80% identity;; ^d^ ICEHo-I, query sequences: 14 MICE core genes of FBG, detection threshold: > 80% identity for > 80% of query sequences in a genomic region ≤35 kb; ^e^ ICEHo-II, query sequences: 5 MICE core genes of SP3615, detection threshold: > 80% identity for > 80% of query sequences in a genomic region ≤20 kb

### Episomal occurrence of ICEHo elements

Nanopore reads of the 23 sequenced *M. hominis* strains were mapped to circularized ICEHo-I (strains FBG and SP10291) and ICEHo-II (SP3615 and SP10291). Reads overlapping the IRR-IRL junction site were only detected in strains FBG (ICEHo-I) and 19791 (ICEHo-II). To detect the presence of episomal ICEHo-I and ICEHo-II with increased sensitivity, a Real time PCR assay, designed to exclusively amplify episomal circularized ICEHo (cICEHo), was employed (see Methods). Application of this cICEHo screening assay to 80 ICEHo-positive isolates from our collection showed that more than two thirds (49/60) of the ICEHo-I- and more than half (27/43) of the ICEHo-II-carrying strains harbour episomal circularized versions of ICEHo-I and -II, respectively (see Additional file 5).

**Fig. 4:**
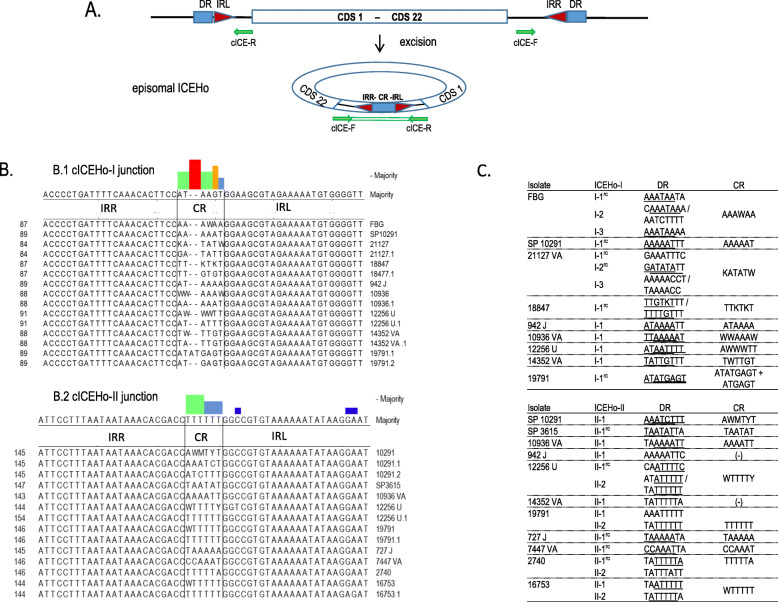
Chromosomal and episomal ICEHo-I and –II. **a** Schematic representation of the excision of a chromosomal integrated ICEHo element to build the circularized episomal cICE-I or -II. ICEHo-flanking left- and right-positioned inverted repeats (IRL and IRR) are represented by red triangles, and the direct repeats (DRs) are represented by blue squares. After excision of ICEHo, direct repeats are fused to the coupling region (CR) in the episomal cICEHo. For the detection of episomal cICEHo-I and -II, specific primer pairs (represented as green arrows) were used that do not lead to an amplification when targeting the chromosomal ICEHo-I and –II in outwards-facing position, and which result in 0.2 (cICE-I) or 0.3 kb (cICE-II) PCR products when targeting the circularized ICEHo (see Methods). **b** Multiple sequence alignments of IRR-IRL junction sequences with the central CR of cICE-I (B.1) and cICE-II (B.2), determined by Sanger sequencing of cICE PCR products. Ambiguity characters in the coupling region (M = A or C; K = G or T; W = A or T; Y = C or T) indicate the presence of minor sequence variants as detected by Mixed Sequence Reader [27]. The respective sequences are listed as *strain*.1 for the major sequence and *strain*.2 for the minor sequence. Coloured bar charts above the consensus sequence, created with the Lasergene software package (DNAStar, Madison, WI), indicate the presence of non-consensus characters (disagreements) in the corresponding column, ranging from red (high frequency of non-consensus characters or gaps) to blue (low frequency of non-consensus characters). **c** List of direct repeats (DR) of the chromosomal ICEHo-I and cICEHo-II copies (I-1 to I-3; II-1 to II-2) detected in the nanopore-sequenced *M. hominis* strains in comparison to the episomal coupling regions (CR). Identical (or comparable) sequences are underlined; if two different regions of one DR are underlined, both regions used for recombination explain the mismatch in CR. IUAPC ambiguity characters in the genomic DR sequences were introduced during the removal of circular contig overlaps (see Methods). I-x^rc^ = the respective ICEHo-element is inserted in a reverse-complementary fashion with respect to the genome assembly, requiring evaluation of the reverse complementary DR

In all whole-genome-sequenced samples, the coupling region (CR) of the episomal ICEs was characterized with Sanger sequencing. In all cases except for cICEHo-I of strain 19791, the detected cICEHO-I and cICEHo-II CR sequences had a length of 6 nucleotides (Fig. 4). The CR of cICEHo-I in strain 19791 consisted of a mixture of six- and eight-nucleotide sequences (ATGAGT and ATATGAGT), with the longer version dominating (see Methods). CR sequences were characterized by a dominance of weak nucleotides (W = A or T) and generally corresponded to the genomic sequences of the IRR−/IRL-flanking direct repeats. The CR of circularized ICEHo-I was typically composed of nucleotides 1–6 of the DR (*n* = 11), less often of nucleotides 3–8 (*n* = 4) or 1–8 (n = 1). The CR of circularized ICHo-II, by contrast, was typically composed of nucleotides 3–8 of the DR (*n* = 7), less often of nucleotides 1–6 (n = 4) or 2–7 (n = 1).

The detection of major and minor CR sequence variants may reflect (i) simultaneous usage of different DR subregions from the same ICEHo element (see underlined sequence regions of DR in Fig. 4.C), (ii) simultaneous observation of multiple ICEHo-I/−II elements with different DR sequences (ICEHo-II of strain 12256 U), (iii) circularisation- or recombination-associated mutagenesis in the circularized ICEHo product, (iv) sequencing error. Of note, minor CR sequence variants were also observed in isolates in which only one ICEHo copy was present (e.g. ICEHo-II of SP10291), and we observed mismatches between CR and the underlying genomic DR sequences in both high quality and draft de novo assembled genomes (e.g. AAAAAA in ICEHo-I of FBG, TTTTGTT in ICEHo-I of 14352VA, and TTTTTT ICEHo-II of 16753). Of note, joint analysis of CR and DR sequences enabled the mapping of circularized ICEHo copies to their respective genomic origins in strains FBG (for ICEHo-I_1 and _3), 21127 (ICEHo-I_2), 19791 (ICEHo-II_2), and 2740 (ICEHo-II_1). In strain 16753, both copies of ICEHo-II were found in circularized form.

## Discussion

Mobile genetic elements play an important role in mediating prokaryotic genome plasticity, often contributing to important phenotypes such as virulence and antibiotic resistance. MGEs can exert their effect by expanding the gene set of the host, or via the disruption of existing genes in the case of integration events. In the present study, we detected and characterized four types of MGEs in clinical isolates of *M. hominis*: ISMhom-1, an insertion sequence; prophage MHoV-1; a *tet*(M)-carrying transposon; and ICEHo-I and -II, two *M. hominis*-specific integrative and conjugative elements.

### MGE insertion patterns

In our study, ISMhom-1 was found exclusively in noncoding chromosomal regions; in other studies, IS element insertions are also reported in MICEs [28] or MICE vestiges [29]. The other types of MGEs detected here were also found to be inserted in coding regions; 2 of 7 detected MhoV-1 insertions led to the interruption of a gene; 6 of 10 ICEHo-I insertion events; and 1 of 2 ICEHo-II insertion events. In more than half of the 11 *M. hominis* genomes assembled to high quality, a chromosomal gene was found to be disrupted by insertion of an MGE. No statistically significant effects were identified during the MGE co-occurrence analysis; the conjecture that ICEHo-I-free isolates may be less susceptible to the entry of other mobile elements [15] was not replicated here.

### ICEHo-I and -II gene content

Horizontal transfer of ICEs from one host to the other is mediated by type IV secretion systems (T4SS), typically comprising the surface-localized pilus, the integral membrane core channel, a protein complex at the cytoplasmic site of the membrane, and ATPases at the cytoplasmic site of the channel [30]. In addition, mobilization and integration of ICEs typically require the presence of a relaxase or integrase enzyme. Genes that participate in the mobilization or conjugation process are referred to as core genes, whereas cargo genes often encode ICE-associated phenotypes of interest, such as resistance [14], metabolic traits [31], or virulence [32]. Characterization of ICEs in mycoplasmas (MICE) has enabled the definition of a MICE core gene set [22], including a mycoplasma-minimized T4SS [22]. ICEHo-II contains a smaller set of MICE core genes than ICEHo-I, but the impact of this on the transfer potential of ICEHo-II remains to be studied. Table 5 shows results of a bioinformatics analysis of putative gene functions, and an integrated view of putative MICE gene functions incorporating results from the literature is shown in Additional file 6. Low levels of homology present significant challenges for the in silico characterization of MICE genes; follow-up experimental studies will be necessary to better characterize the functions of ICEHo-I and -II genes.

**Table 5:** Sequence-based characteristics of ICEHo-II encoded proteins

MICE-/ICEHo-	SP3615_ ICEHo-II	SP10291_ ICEHo-II	Homologues proteins	Species	Accession number	TMHMM v.2.0	SignalP v. 5.0	Motif	Reference of motif	DEPP S - T - Y^c^	Protein length	Query cover	E-value	Identity
CDS	KGPEAEHF^a^	HPAMDCMO^a^	BLASTp /[PSI-BLAST^b^]							(x/y)	(AA)	(%)		(%)
MICE_ CDS1	_00500	_00016	ICEF-IA CDS1	M. fermentans	AY168953.1	–	–	rpoC2	CHL00117	2/21–0/10–0/12	252	88.89	4.22E-03	36.60
MhoM	_00499	_00017	hypothetical protein	M. hominis 4235	WP_158532045.1	–	–	–	–	6/7–1/2–1/6	108	90.00	1.00E-58	87.76
MhoM	_00498	_00018	hypothetical protein	M. hominis 4235	WP_158532045.1	–	–	–	–	1/6–1/4–2/5	97	89.00	1.00E-21	48.31
MhoN	_00497	_00019	hypothetical protein	M. hominis PL5	WP_044329953.1	–	–	–	–	3/13–1/1–0/9	157	21.00	3.30E-01	61.76
MhoO	_00496	_00020	hypothetical protein	M. primatum	WP_029513615.1	–	–	–	–	0/8–0/8–0/20	217	64.98	2.00E-14	37.59
MhoP	_00495	_00021	variable lipoprotein	M. arginini	WP_129694644.1	1	II	Lipoprot. 7	pfam01540	0/14–0/13–1 / 14	272	45.96	3.86E-09	33.60
MhoQ	_00494	_00022	hypothetical protein	I. bacterium	OIO22386.1	–	–	AcrB	COG0841	0/4–0/3–0/2	93	59.00	7.40E-02	38.18
MhoR	_00493	_00023	response regulator ^b^	C. estertheticum	WP_152751962.1	–	–	–	–	1/10–0/2–0/2	102	87.00	9.90E-01	29.59
MhoS	_00492	_00024	hypothetical lipoprotein	M. phocicerebrale	WP_116171775.1	–	II	–	–	5/27–1/21–2/22	415	99.00	1.00E-109	45.93
			BspA leu-rich repeat surface protein	*M. bovis*	WP_141790766.1			–	–			100.00	5.00E-101	13.17
MhoT	_00491	_00025	hypothetical protein	M. phocicerebrale	WP_116171528.1	2	–	–	–	0/7–0/9–0/1	98	94.00	1.00E-19	72.04
MhoU	_00490	_00026	hypothetical protein	M. phocicerebrale	WP_116171527.1	2	–	–	–	0/3–0/5–0/5	85	97.00	1.00E-14	42.86
MICE_ CDS16	_00489	_00027	hypothetical protein	M. phocicerebrale	WP_116171526.1	7	–	smc_procA	TIGR02169	6/36–2/18–2/20	536	98.00	0.0	56.96
			ICEF_-A CDS16 ^b^	M. fermentans	AAN85226.1							67.35	1.00E-34	14.20
MICE_ CDS17	_00488	_00028	hypothetical protein	M. phocicerebrale	WP_116171521.1	3 / 2	–	TraC_F-type	TIGR02746	0/58–5/49–1/35	885	99.00	0.0	65.34
			TraE/TraC^b^	Mycoplasma spp.	WP_013526664.1							99.00	0.0	37.02
MICE_ CDS19	_00487	_00029	DNA polymerase III	M. phocicerebrale	WP_116171520.1	2	I	polC	PRK00448	15/112–7/113–5/99	1821	99.00	0.0	44.38
			ICE-M CDS19^b^	M. mycoides	CBW53951.1							82.00	2.00E-123	26.29
MICE_ CDS22	_00486	_00030	hypothetical protein	M. phocicerebrale	WP_116171517.1	–	–	UPF0236	pfam06782	0/21–0/12–0/24	387	100.00	8.00E-125	52.20
			ISLre2 transposase ^b^	H. hemicellulosilytica	WP_125474456.1							99.00	9.00E-161	19.11

- = no detection; ^a^ number of the respective ICEHo_II ORFs in SP3615_(KGPEAEHF_00500 to _00468) and SP10291 (HPAMDCMO_00016 to _00030) according to Additional file 7; ^b^ homologous proteins identified by PSI-BLAST; ^c^ DEPP predicted phosphorylation sites (x) compared to total numbers of possible phosphorylation sites (y) of *S* serine, *T* threonine, *Y* tyrosine

### Circularization is likely indicative of ICEHo-I and -II transfer potential

For ICEs, excision and circularization represent key steps in the mobilization process [33]. We used a specifically developed PCR assay to demonstrate the presence of ICEHo-I and -II in their episomal circularized forms across many isolates in our screened cohort. The detection of circularized ICEHo-I and -II demonstrates the first step in the potential horizontal transfer of these elements and indicates that ICEHo-I and -II likely retain their mobile potential. Interestingly, we also detected the presence of minor sequence variants in the coupling region of circularized ICEHo-I and -II elements that could not readily be explained based on the respective genomic DR regions. Follow-up studies to confirm the existence of these minor CR sequence variants and to characterize their potential functional are an important direction for future work.

## Conclusions

Nanopore sequencing enabled the characterization of mobile genetic elements and the identification of ICEHo-II, a novel MICE element of *M. hominis*. Our characterization provides a starting point for the elucidation of the function of the ICEHo-I and -II cargo genes and their phenotypic impact, in particular with respect to a potential impact on the pathogenicity of this genetically heterogeneous human facultative pathogen.

## Methods

### M. hominis strains

*M. hominis* strains were isolated from human specimens. Strains FBG, 8958 and 2539 were part of a collection of clinical *M. hominis* strains, created at the Institute of Pathology of the Johannes Gutenberg University Mainz, Germany, and transferred in 1988 to the Institute of Med. Microbiology and Hospital Hygiene at the Medical Faculty of the Heinrich-Heine-University of Duesseldorf; strains 475 and A136 derived from the Institute of Microbiology, University of Veterinary Medicine Vienna, Austria; strains SS10 and SS25 from the Institute for Specific Prophylaxis and Tropical Medicine, Centre for Pathophysiology, Immunology and Infectiology, Medical University of Vienna, Austria; and strains SP3615, VO31120, SP10291 and SP2656 were part of the strain collection of our institute, collected within the last 10 years. FBG, 8958 and 2539 were isolated from women; only for isolate 8958, the donor’s age (64) and strain location (vaginal) are known. Strain 475 was isolated from vaginal specimen; A136 and SP3615 were isolated from placenta after preterm birth; VO31120 was isolated from pleura of a patient with pneumonia; SP10291 was isolated from brain material after cerebral infarction; SP2565 was derived from blood culture of a patient in NHL remission [34]; SS10 and SS25 were isolated from in vitro cultured *T. vaginalis* (as endosymbionts). Protozoa were isolated from women affected by acute trichomoniasis respectively in 1996 and 1999, at the Department of Biomedical Sciences, University of Sassari, Italy [35]. All other *M. hominis* strains were taken from the strain collection of our institute in Duesseldorf, lacking information about associated diseases.

### *M. hominis* culturing and genomic DNA preparations

*M. hominis* strains were cultivated in arginine-medium as described in detail previously [36]. Genomic DNA of the strains was isolated by the use of the QIAamp Blood and Tissue kit (Hilden, Germany) following the tissue protocol with minor modifications as published [37]. Concentration of genomic DNA was measured by Invitrogen Qubit 4 Fluorometer Qubit and its quality verified spectrophotometrically by NanoDrop 1000 Spectrophotometer and on a Fragment Analyzer System (Agilent, Santa Clara, CA USA) with method DNF-464-33 for high sensitivity large fragment 50 kb analysis.

### Whole genome sequencing and assembly of *M. hominis* strains

#### Generation of short-read sequencing data

Short-read Illumina sequencing was carried out for 11 isolates. Sequencing libraries were prepared according to the manufacturer’s instructions and sequenced on the MiSeq platform with 2 × 300 bp or 2 × 250 bp paired-end sequencing protocols (Table 1).

#### Nanopore sequencing and assembly

22 *M. hominis* strains were sequenced on a MinION MK1B device. Sequencing libraries from quality-controlled genomic DNA were prepared according to the manufacturer’s instructions, employing the rapid (2 samples), ligation-based (1 sample), and barcoded ligation-based (19 samples) protocols (Table 1). Basecalling and demultiplexing were carried out with MinKNOW (basecalling only) and Albacore (basecalling and demultiplexing).

Canu [17] 1.6 (with parameters -genomeSize = 1 m -nanopore-raw) was used for the assembly of the generated long-read data, yielding one large contig for each sample but one. Two smaller contigs in the assemblies of samples SS25 and SP2565 had only spurious read support as reported by Canu and were removed from the assembly. To generate “high quality” assemblies for the samples for which short-read data were available, the assemblies of the first 10 samples (Table 1) were polished with Nanopolish [38] 0.8.4, circular overlaps at the ends of contigs were removed, and orientation to the PG21 type strain genome was carried out. Two rounds of Pilon [39] 1.22 were used for further polishing based on short reads for each of the “high-quality” assemblies. Finally, short-read data were aligned against the Pilon-polished assemblies; GATK [40] 3.7 (with parameters -T HaplotypeCaller -ploidy 1) was used to call variants; and reference alleles were substituted with variant alleles whenever the reference allele frequency, measured via samtools mpileup -q0 -Q10 [41], was ≤10%. All short-read alignments were generated with bwa mem [42] 0.7.15-r1140. The genome structure of the generated assemblies was examined with nucmer [43] and the effectiveness of the polishing strategy was assessed by visually screening for potential base errors in IGV [44]. For the remaining 12 samples for which no short-read data were available, Nanopore-only based assemblies (referred to as “draft assemblies”) as produced by Canu were only used to characterize the MGEs contain within them. Draft genomes were oriented to the PG21 type strain and the circular contig overlap region was substituted with a consensus sequence of the two underlying overlaps, computed with SeqMan Version 6.0 (DNASTAR. Madison, WI). Of note, ambiguities in the computed consensus were represented using IUPAC ambiguity characters.

To further improve sequence quality for a triplicate repeat (later identified as ICEHo-I) identified by our inspection strategy in the genome of sample FBG, we applied a modification of the GATK-based polishing strategy described above. First, all short reads aligned to any of the three copies of the repeat in the genome of sample FBG were extracted. Second, for the three assembled repeat sequences independently, the complete set of extracted reads was aligned against the individual instance of the repeat and variants were called with GATK (using -ploidy 3). Finally, reference alleles were substituted with variant alleles at homozygous variant positions with reference allele frequency ≤ 10%. A manuscript describing a generalization of our approach and presenting a stand-alone software implementation is currently under preparation.

#### PacBio sequencing and assembly

Library preparation for long-read sequencing of *M. hominis* isolate SP3615 on the Sequel system was carried out with the SMRTbell Template Prep Kit 1.0 and the Sequel Binding and Internal Control Kit 2.1, using the “Greater than 10kb Template Protocol” and 10 h movie time. Assembly was carried out with HGAP4 (SMRT Link Version 5.1.0.26412) [18] and polished with Arrow. Orientation and removal of circular overlaps were carried out as described above,. Visual inspection was used to confirm the quality of the generated assembly.

### qPCR

Oligonucleotides used in qPCRs were designed using Probefinder (Roche Applied Science) (https://qpcr.probefinder.com). Primers are listed in Table 6.

**Table 6:** Primers used

Gene	qPCR primer	Sequence (5′-3′)	Amplicon length (nt)	PCR protocol
ICEHo-I_*dcm*	463_F	CACGGATCTCCTTGTCAAGAT		
	463_R	TGTTTCCCACAATAAACTACTTCG	91	1
ICEHo-I_CDS5	462_F	AGAAGATTTGTCAAAAACTCCTAAAGA		
	462_R	ACCACTTTGTGCTTCGGCTAA	64	1
ICEHo-I_CDS14	474_F	CCAAATCCTTCAAACCCAACT		
	474_R	TCTGGTTTAACTTCAGGGGTTG	62	1
ICEHo-I_CDS16	476_F	GCAATTGCTTTTGTTGGAAGT		
	476_R	CTGATCTTGCTCCAGACATAGC	73	1
ICEHo-I_CDS17.1	477_F1	GATTTTGTGCCGTCATCGTA		
	477_R1	TTTAAAATGGCAGGATTATCAGG	67	1
ICEHo-I_CDS17.2	477_F2	RAAAATATTTGCAAGAACATAACATTA		
	477_R2	ATTTTCTAACCGTTTTTGTCATTT	165	1
ICEHo-II_CDS17	17-II_F	CCCAATAAATCCGATAGCATTA		
	17-II_R	GTTCCCAACACTAACATTCCTC	84	1
circular ICEHo-I	cICE_I-F	GCGGGCGCGTAGAGCAT		
	cICE_I-R	TATTTGGAATTAACCCCACATTTT	185	2
circular ICEHo-II	cICE_II-F	CAAAATTCAGATTAATTACTAATAAACAAA		
	cICE_II-R	AGAGCATGAGCAAGAAAAAAAGTA	290	2
hitA	*hit*A_F	TTGAGGCACAGCAATAGC		
	*hit*A_R	AAGGCTTAGGTAAGGAATTGATTAG	81	1
*gap*	*gap*_F	GCAGGCTCAATATTTGACTCACT		
	*gap*_R	GATGATTCATTGTCGTATCATGC	95	1

qPCR assays were carried out in a total volume of 25 μl consisting of 1 × MesaGreen MasterMix, 5 mM MgCl_2_, Amperase, 300 nM of each primer and 2.5 μl of genomic DNA or cDNA solution, which was derived from 20 ng RNA. Thermal cycling conditions were as follows: 1 cycle at 50 °C for 10 min, 1 cycle at 95 °C for 5 min followed by 45 cycles of 95 °C for 15 s and 60 °C for 1 min (protocol 1) or 1 cycle at 95 °C for 5 min followed by 35 cycles of 95 °C for 15 s, 30 s 55 °C and 60 °C for 45 s (protocol 2). The product was than heated from 65 °C to 95 °C with an increment of 0.5 °C/15 s and the plate read for melt curve analysis to check the identity of the amplicon. Each sample was analysed in duplicate. Cycling, fluorescent data collection and analysis were carried out on a CFX-Cycler of BioRad Laboratories (Munich, Germany) according to the manufacturer’s instructions.

### ICEHo qPCR screening assay

Real time PCR (qPCR) was used to screen for the presence of ICEHo-I and -II. For ICEHo-I, qPCR was used to determine the presence of MICE core genes CDS5, − 14, − 16, − 17, and of the ICEHo-I specific *dcm* gene. For ICEHo-II, qPCR was used to determine the presence of a conserved region of the ICEHo-II CDS17 gene. Utilized primers are listed in Table 6. Ct values were interpreted relative to the chromosomal *M. hominis*-specific *hit*A gene [45, 46] (see Additional file 5), with ∆Ct values (defined as Ct (ICEHo gene X) – Ct (*hit*A)) ≥ 10 classified as negative, and ∆Ct-values < 10 classified as positive. The utilized ∆Ct value threshold of 10 was determined based on strains FBG (ICEHo-I), SP13615 (ICEHo-II), and SP10291 (ICEHo-I and –II) as positive controls, and ICEHo-free strains PG21, 8958, 2539, SP2565, SS10, and VO31120 as negative controls. For ICEHo-I, isolates in which all PCRs were positive were classified as unambiguously positive; isolates in which at least two PCRs were positive were classified as positive; and isolates in which 0 or 1 PCRs were positive were classified as negative. With the chosen threshold values and decision algorithm, assembly- and qPCR-based results were in perfect agreement for the sequenced strains (Additional file 5).

### qPCR screening for episomal circularized ICEHo (cICEHo)

Real time PCR (qPCR) was used to screen for the presence of ICEHo-I and -II in their episomal circularized forms, utilizing outwards-facing primer pairs (cICE_I-F/_I-R and cICE_II-F/_II-R; see Table 6). For ICEHo-I, these primers targeted the conserved untranslated ICEHo-I regions 266 bp downstream of CDS22 (cICE_I-F) and 175 bp upstream of CDS1 (cICE_I-R), leading to PCR products of 0.2 kb in case of episomal circularisation. For ICEHo-II, they targeted the conserved untranslated ICEHo-II regions just downstream of CDS22 (cICE_II-F) and 152 bp upstream of CDS1, leading to cICE-II PCR products of 0.3 kb in case of episomal circularisation (see Fig. 4.A). In the whole-genome-sequenced samples, all cICE amplification products were sequenced with Sanger sequencing, confirming cICE detection results through the detection of a valid IRR-IRL junction and coupling region (CR) in all but two cases with high qPCR Ct values (33 and 31; Additional file 5). At higher cycle counts (> 30), SybrGreen-based qPCRs are known to be prone to false-positives due to the generation of primer dimers or mispriming to imperfect binding sites. For the wider cohort of samples that were only screened with qPCR, all cICE-PCRs with Ct values > 30 were thus classified as negative, unless Sanger sequencing of the PCR product proved the presence of a CR region in the amplification product (Additional file 5). Major and minor CR sequence variants were detected by applying the algorithm Mixed Sequence Reader [27] to the Sanger chromatogram data.

### Annotation and bioinformatic analysis of *M. hominis* genomes

Prokka [20] was used to annotate the assembled genomes. PHAST (**PHA**ge **S**earch **T**ool) (http://phast.wishartlab.com/) was used to identify and annotate prophage sequences [47]. BLAST Microbes (https://BLAST.ncbi.nlm.nih.gov/BLAST.cgi) was used for detection of homologous genes and plasmids. Multiple sequence alignments were calculated by using Genious Pro (vers. 5.5.8) and MegAlign version 6.0 of the Lasergene software package (DNAStar, Madison, WI). Genome alignments illustrating gene gain, loss and rearrangement were done with Mauve [21]. The Phyre2 web portal was used for protein modelling, prediction and analysis [48]; RADAR for detection and alignment of repeats in protein sequences ( [49] https://www.ebi.ac.uk/Tools/services/web_radar/toolform.ebi); MEME for discovering novel, ungapped motifs (recurring, fixed-length patterns) ( [50] http://meme-suite.org/tools/meme); Disorder Enhanced Phosphorylation Predictor (DEPP) (http://www.pondr.com/cgi-bin/depp.cgi).

### Statistical programs used

Statistical tests were performed in Stata 14 (StataCorp, TX). Associations of presence of different MGEs were assessed by Chi-square test, associations of abundance by Spearman’s rank correlation.

## Supplementary Information

**Additional file 1. **Homology of CDS6. Deduced protein sequences of CDS6 derived from *M. hominis* strains FBG (BHBFJMJE_00471), SP10291 (HDENHCDK_00553), TO0613 (WP_036439043.1), PL5 (WP_036439043.1), and 4788 (WP_036439043.1) and of *M. fermentans* strains M64 (ADV34390.1 (I) and ADV34456.1(II)) and PG18 (ICEF-IA (AAN85216.1 (IA)) and ICEF-II-A (AAN85259.1 (II-A)). Two chromosomal proteins of *M. hominis* PG21 (MHO_0070 (CAX37141.1) and MHO_1280 (CAX37262.1)) were used as unrelated ICE-outliners in ClustalW-based Multiple Sequence Alignment. A. Phylogenetic tree; B. Percent amino acid identities and divergences; and C. Multiple Sequence Alignment of CDS6 encoded proteins. Identical amino acids are marked in green, isofunctional amino acids marked in yellow.

**Additional file 2.** Clustering of MhoH and MhoJ. MhoH and MhoJ proteins of FBG, SP10291, PL5 and TO0613 were clustered in multiple sequence alignment using Clustal W and divided in five subgroups (MhoH1 to MhoH3 and MhoJ1 and MhoJ2) according to their phylogenetic relationship (A.). All MhoH and MhoJ proteins carried the TAL-effector motif in the C-terminal part (B.). Percent amino acid identities and divergences are shown in C.).

**Additional file 3.** Phylogeny of MhoM and CDS11. MhoM encoded proteins of ICEHo-I and -II elements of strains FBG, SP10291, PL5, 4788 and TO0613 were clustered with the respective CDS11 genes in multiple sequence alignment using Clustal W. A.) Phylogenetic tree of MhoM and CDS11 encoded proteins. B.) Percent identities and divergences of MhoM and CDS11 proteins.

**Additional file 4.** ICEHo locations in draft genomes. Positions and gene presence patterns of ICEHo-I and -II in draft de novo assemblies of 12 *M. hominis* strains sequenced only with Nanopore. ICEHo positions in the draft assemblies were determined by aligning the sequences of ICEHo-I of strain FBG and of ICEHo-II of strain SP3615 to the draft assemblies. The additional columns show the homology (nucleotide identity %) between the genes present in the draft assembly ICEHo elements and the genes present in ICEHo-I of FBG and the genes present in ICEHo-II of SP3615 (gene order and names correspond to Fig. 3).

**Additional file 5.** qPCR data of ICEHo and cICE. Ct and ∆Ct values for ICEHo-I, −II and cICE detection, as well as ICEHo-I and -II status based on the assembled genomes, and confirmatory detection of cICE by Sanger sequencing.

**Additional file 6.** ICEHo-I and ICEHo-II putative gene functions. ICEHo-I and -II putative gene functions, based on bioinformatics analyses (see main text) and literature review.

**Additional file 7.** M. hominis genomes in GB.

## Data Availability

Genome sequences of strains LBD-4 (acc.-no. CP009652.1), PG21 (acc.-no. FP236530.1), SPROTT (acc.-no. CP011538.1), TO0613 (acc.-no: CP033021.1) and contigs of strain PL5 (acc.-nos: JRXA01000001.1 - JRXA010000016.1) were downloaded from NCBI (https://www.ncbi.nlm.nih.gov/nuccore/). All raw sequencing data and high-quality assemblies are made available under BioProject PRJNA429440; all draft assemblies are available at OSF (DOI: 10.17605/OSF.IO/CZRBT). Generated genome sequences in FASTA format are available at NCBI. Generated genome sequences in GenBank format and annotated using this publication’s annotation terminology are provided as an Additional file 7.

## Abbreviations

CDS: CoDing sequence
CR: Coupling Region
DEPP: Disorder enhanced phosphorylation predictor
DR: Direct repeat
ICE: Integrative and conjugative element
ICEHo: Integrative and conjugative element of *M. hominis*
MICE: Mycoplasmal integrative and conjugative element
MGE: Mobile genetic element
MTase: Methyltransferase
PHAST: PHAge search tool
qPCR: Real time quantitative PCR
R-M system: Restriction-modification system
SRP: Signal recognition particle
T4SS: Type IV secretion system
TAL: Transcription activator-like
