# Supplementary material for: Characterisation of mobile genetic elements in Mycoplasma hominis with the description of ICEHo-II, a variant mycoplasma integrative and conjugative element
Source: Mob DNA. 2020 Nov 7;11:30. doi: 10.1186/s13100-020-00225-9 (PMC7648426; doi:10.1186/s13100-020-00225-9)
Supplement: Supplementary file 1 — Additional file 1. Homology of CDS6. Deduced protein sequences of CDS6 derived from M. hominis strains FBG (BHBFJMJE_00471), SP10291 (HDENHCDK_00553), TO0613 (WP_036439043.1), PL5 (WP_036439043.1), and 4788 (WP_036439043.1) and of M. fermentans strains M64 (ADV34390.1 (I) and ADV34456.1(II)) and PG18 (ICEF-IA (AAN85216.1 (IA)) and ICEF-II-A (AAN85259.1 (II-A)). Two chromosomal proteins of M. hominis PG21 (MHO_0070 (CAX37141.1) and MHO_1280 (CAX37262.1)) were used as unrelated ICE-outliners in ClustalW-based Multiple Sequence Alignment. A. Phylogenetic tree; B. Percent amino acid identities and divergences; and C. Multiple Sequence Alignment of CDS6 encoded proteins. Identical amino acids are marked in green, isofunctional amino acids marked in yellow. [file 13100_2020_225_MOESM1_ESM.pdf]

CDS6-Homologues

A. Phylogenetic Tree

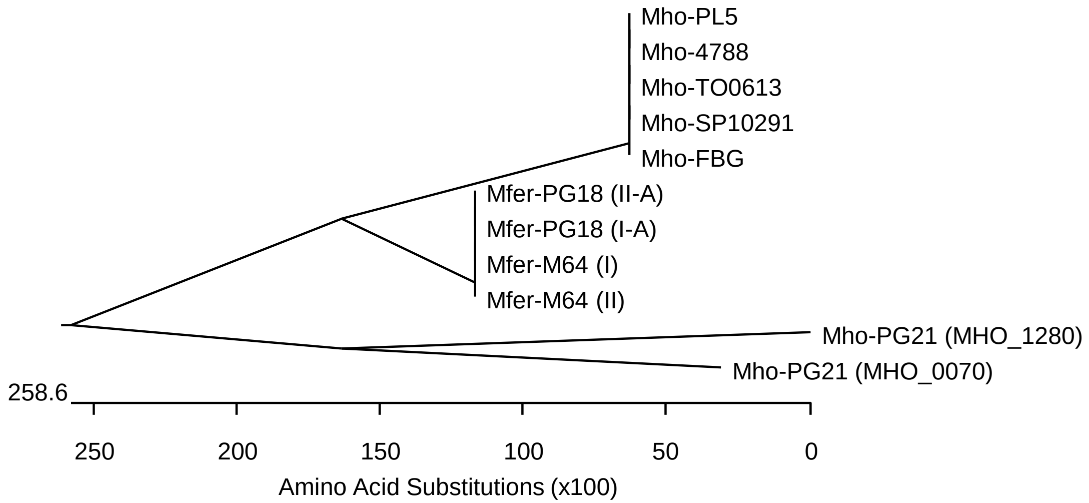

B. Sequence Distances

Percent Identity (upper triangle); Divergence (lower triangle)

	1	2	3	4	5	6	7	8	9	10	11		
**1**		100.0	100.0	100.0	32.3	32.3	32.3	32.3	32.3	11.3	14.5	**1**	Mfer-PG18 (II-A)
**2**	0.0		100.0	100.0	32.3	32.3	32.3	32.3	32.3	11.3	14.5	**2**	Mfer-PG18 (I-A)
**3**	0.0	0.0		100.0	32.3	32.3	32.3	32.3	32.3	11.3	14.5	**3**	Mfer-M64 (I)
**4**	0.0	0.0	0.0		32.3	32.3	32.3	32.3	32.3	11.3	14.5	**4**	Mfer-M64 (II)
**5**	146.6	146.6	146.6	146.6		100.0	100.0	100.0	100.0	11.1	11.1	**5**	Mho-FBG)
**6**	146.6	146.6	146.6	146.6	0.0		100.0	100.0	100.0	11.1	11.1	**6**	Mho-SP10291
**7**	146.6	146.6	146.6	146.6	0.0	0.0		100.0	100.0	11.1	11.1	**7**	Mho-TO0613
**8**	146.6	146.6	146.6	146.6	0.0	0.0	0.0		100.0	11.1	11.1	**8**	Mho-PL5
**9**	146.6	146.6	146.6	146.6	0.0	0.0	0.0	0.0		11.1	11.1	**9**	Mho-4788 (MhoE)
**10**	430.0	430.0	430.0	430.0	430.0	430.0	430.0	430.0	430.0		16.7	**10**	Mho-PG21 (MHO_1280)
**11**	339.0	339.0	339.0	339.0	447.0	447.0	447.0	447.0	447.0	296.0		**11**	Mho-PG21 (MHO_0070)
	**1**	**2**	**3**	**4**	**5**	**6**	**7**	**8**	**9**	**10**	**11**		

C. Multiple Sequence Alignment

```
                  10                  20                  30                  40                  50                  60
1 M K R Y F Y R I R - V K K N L E S K K T K S Y A V C S - N F N E A D K I A N E I I E K G D Y G - - W V Q V E R V E K I K H Y F K E V   Mfer-PG18 (II-A)
1 M K R Y F Y R I R - V K K N L E S K K T K S Y A V C S - N F N E A D K I A N E I I E K G D Y G - - W V Q V E R V E K I K H Y F K E V   Mfer-PG18 (I-A)
1 M K R Y F Y R I R - V K K N L E S K K T K S Y A V C S - N F N E A D K I A N E I I E K G D Y G - - W V Q V E R V E K I K H Y F K E V   Mfer-M64 (I)
1 M K R Y F Y R I R - V K K N L E S K K T K S Y A V C S - N F N E A D K I A N E I I E K G D Y G - - W V Q V E R V E K I K H Y F K E V   Mfer-M64 (II)
1 M S K Y F Y R L R G S Q S T G K N A K T Y S F G S A Q - Q Y N K A L I M A Q D A I S K N Q F K - - E V Q I E R I Q V I K I I K Q N S   Mho-FBG
1 M S K Y F Y R L R G S Q S T G K N A K T Y S F G S A Q - Q Y N K A L I M A Q D A I S K N Q F K - - E V Q I E R I Q V I K I I K Q N S   Mho-SP10291
1 M S K Y F Y R L R G S Q S T G K N A K T Y S F G S A Q - Q Y N K A L I M A Q D A I S K N Q F K - - E V Q I E R I Q V I K I I K Q N S   Mho-TO0613
1 M S K Y F Y R L R G S Q S T G K N A K T Y S F G S A Q - Q Y N K A L I M A Q D A I S K N Q F K - - E V Q I E R I Q V I K I I K Q N S   Mho-PL5
1 M S K Y F Y R L R G S Q S T G K N A K T Y S F G S A Q - Q Y N K A L I M A Q D A I S K N Q F K - - E V Q I E R I Q V I K I I K Q N S   Mho-4788 (MhoE)
1 M P K M K T K S G - L K K R I S I T E S G K V K R G Q - A F R S H L A Q N K T T K Q K R H A R - - K A T T L S S S D F K R Y K E       Mho-PG21 (MHO_1280)
1 M P G R D Q L T G - Q K A L S G N K R S H A L N T A K R T F D L N L Q K V T V L T E T G S K K T V R V T A K N A R T L K K Y G L V A   Mho-PG21 (MHO_0070)
```
