# Supplementary figures and images for: Characterisation of mobile genetic elements in Mycoplasma hominis with the description of ICEHo-II, a variant mycoplasma integrative and conjugative element
Source: Mob DNA. 2020 Nov 7;11:30. doi: 10.1186/s13100-020-00225-9 (PMC7648426; doi:10.1186/s13100-020-00225-9)

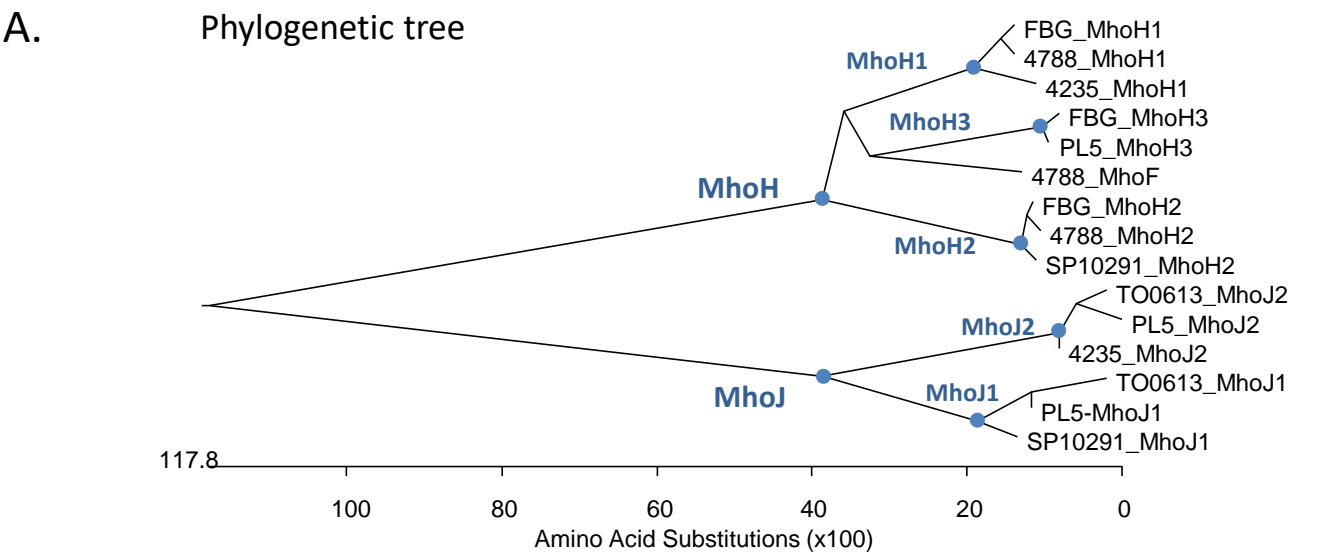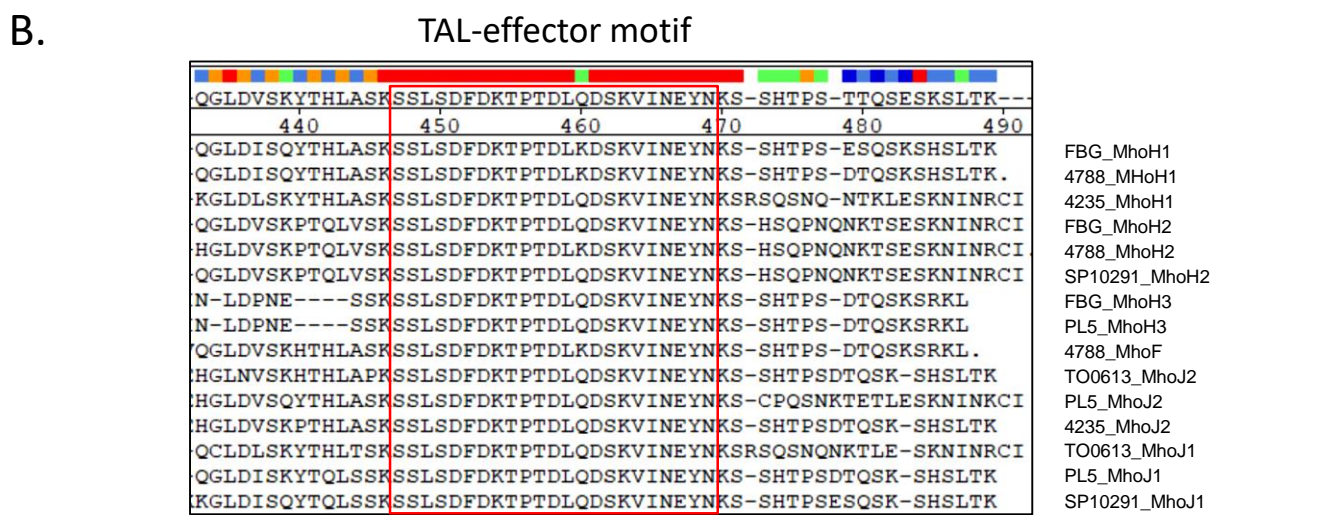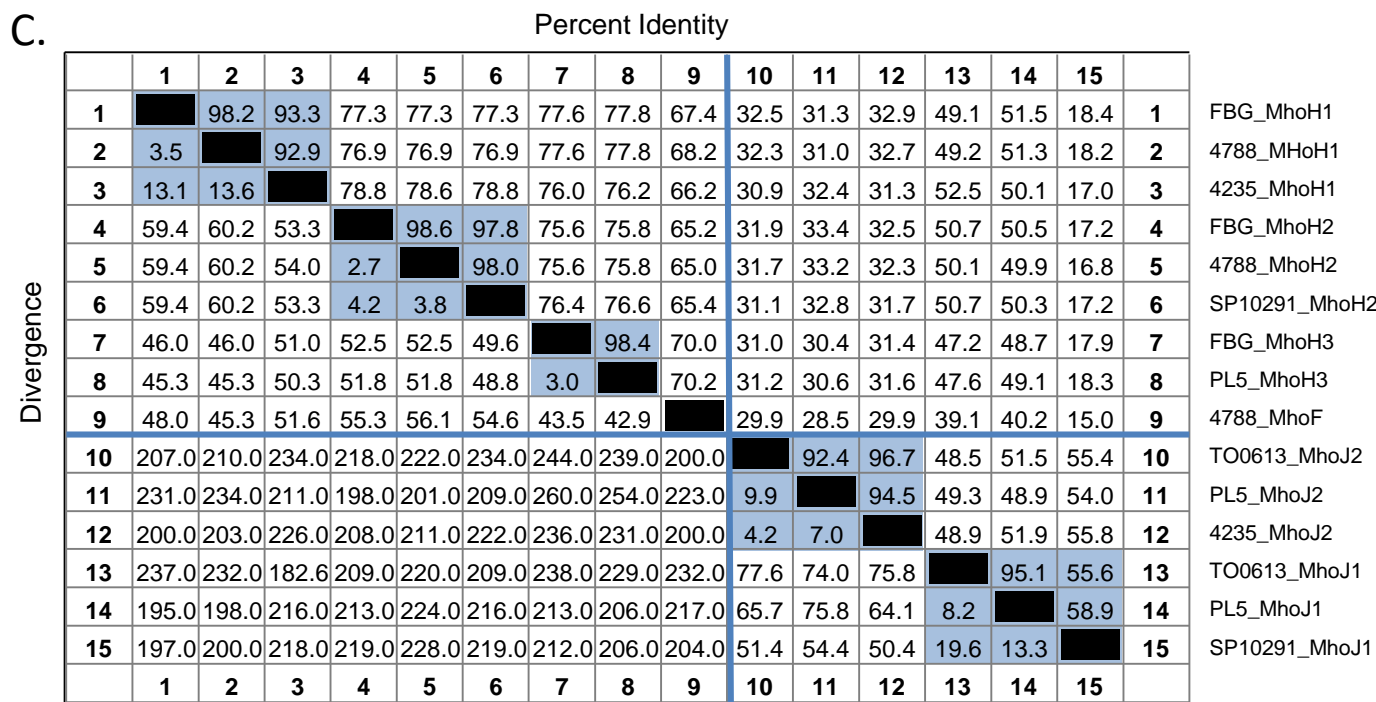

Supplement: Supplementary file 2 — Additional file 2. Clustering of MhoH and MhoJ. MhoH and MhoJ proteins of FBG, SP10291, PL5 and TO0613 were clustered in multiple sequence alignment using Clustal W and divided in five subgroups (MhoH1 to MhoH3 and MhoJ1 and MhoJ2) according to their phylogenetic relationship (A.). All MhoH and MhoJ proteins carried the TAL-effector motif in the C-terminal part (B.). Percent amino acid identities and divergences are shown in C.). [file 13100_2020_225_MOESM2_ESM.pdf]
