# Supplementary material for: Characterisation of mobile genetic elements in Mycoplasma hominis with the description of ICEHo-II, a variant mycoplasma integrative and conjugative element
Source: Mob DNA. 2020 Nov 7;11:30. doi: 10.1186/s13100-020-00225-9 (PMC7648426; doi:10.1186/s13100-020-00225-9)
Supplement: Supplementary file 3 — Additional file 3. Phylogeny of MhoM and CDS11. MhoM encoded proteins of ICEHo-I and -II elements of strains FBG, SP10291, PL5, 4788 and TO0613 were clustered with the respective CDS11 genes in multiple sequence alignment using Clustal W. A.) Phylogenetic tree of MhoM and CDS11 encoded proteins. B.) Percent identities and divergences of MhoM and CDS11 proteins. [file 13100_2020_225_MOESM3_ESM.pdf]

A.

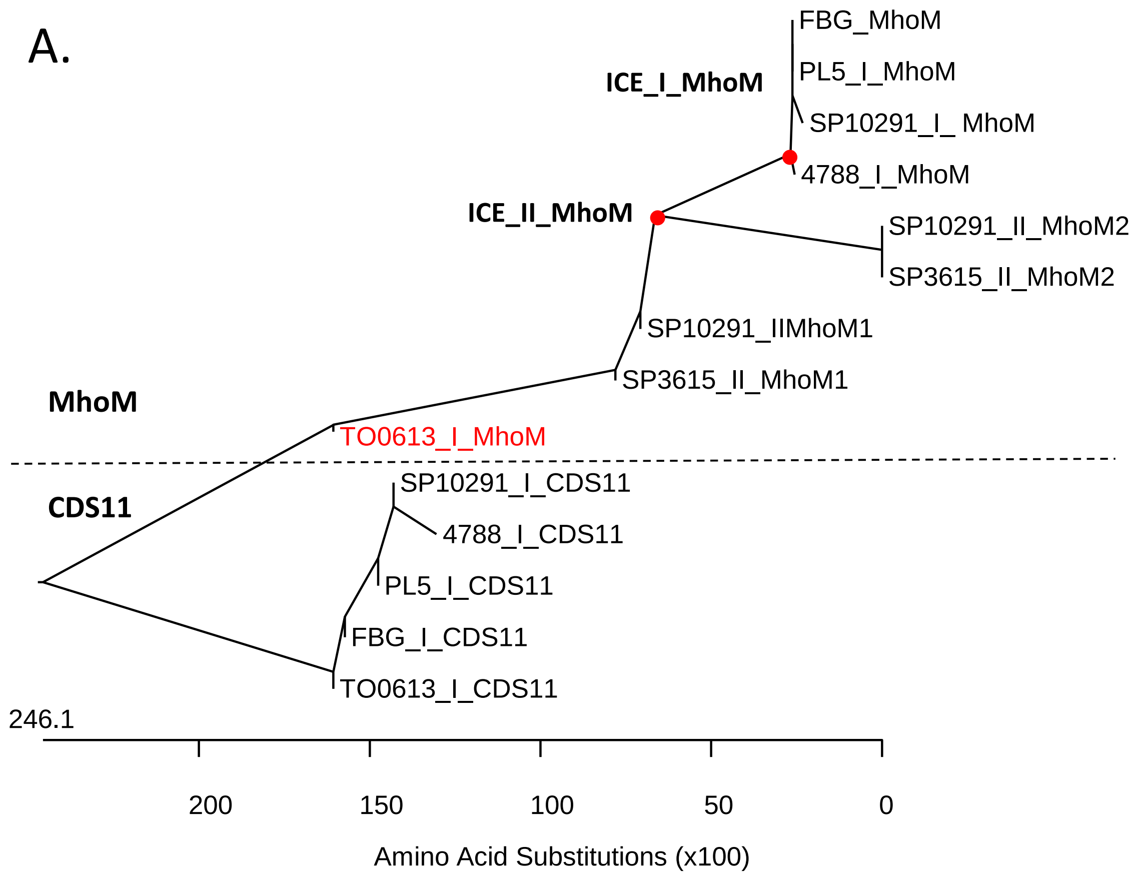

B.

Percent Identity

	1	2	3	4	5	6	7	8	9	10	11	12	13	14		
1		18.8	97.0	18.0	53.4	30.1	53.4	30.1	31.6	19.5	100.0	18.0	98.5	16.5	1	FBG_I_MhoM
2	260.0		9.5	92.1	11.1	6.3	11.5	6.3	22.2	95.6	9.9	98.8	9.9	92.5	2	FBG_I_CDSF11
3	3.1	271.0		17.3	52.6	29.3	52.6	29.3	32.3	18.8	97.0	17.3	95.5	15.8	3	SP10291_I_MhoM
4	271.0	8.4	282.0		10.7	6.0	11.1	6.0	21.8	90.5	9.5	92.5	9.5	92.1	4	SP10291_I_CDS11
5	41.2	188.0	42.8	196.2		48.1	97.2	48.1	60.2	25.0	65.7	25.0	64.8	22.2	5	SP10291_II_MhoM1
6	106.9	296.0	110.7	315.0	70.7		51.5	100.0	50.5	15.5	41.2	15.5	41.2	15.5	6	SP10291_II_MhoM2
7	41.2	180.3	42.8	188.0	2.8	75.8		46.3	58.3	25.0	65.7	25.9	64.8	23.1	7	SP3615_II_MhoM1
8	106.9	296.0	110.7	315.0	70.7	0.0	75.8		50.5	15.5	41.2	15.5	41.2	15.5	8	SP3615_II_AMhoM2
9	104.2	104.9	100.8	107.6	51.6	74.4	55.4	74.4		43.7	31.1	43.7	31.1	39.3	9	TO0613_I_MhoM
10	250.0	4.5	260.0	10.2	196.2	315.0	196.2	315.0	97.4		10.3	96.0	10.3	90.5	10	TO0613_I_CDS11
11	0.0	260.0	3.1	271.0	41.2	106.9	41.2	106.9	104.2	250.0		18.0	98.5	16.5	11	PL5_I_MhoM
12	271.0	1.2	282.0	8.0	196.2	315.0	188.0	315.0	97.4	4.1	271.0		9.5	92.9	12	PL5_I_CDS11
13	1.5	260.0	4.7	271.0	42.8	106.9	42.8	106.9	104.2	250.0	1.5	271.0		16.5	13	4788_I_MhoM
14	296.0	8.0	309.0	8.4	218.0	315.0	209.0	315.0	113.2	10.2	296.0	7.5	296.0		14	4788_I_CDS11
	1	2	3	4	5	6	7	8	9	10	11	12	13	14		
